# Expert-level detection of acute intracranial hemorrhage on head computed tomography using deep learning

**DOI:** 10.1073/pnas.1908021116

**Published:** 2019-10-21

**Authors:** Weicheng Kuo, Christian Hӓne, Pratik Mukherjee, Jitendra Malik, Esther L. Yuh

**Affiliations:** ^a^Electrical Engineering and Computer Sciences, University of California, Berkeley, CA 94720;; ^b^Department of Radiology and Biomedical Imaging, University of California, San Francisco, CA 94107

**Keywords:** intracranial hemorrhage, head computed tomography, radiology, deep learning

## Abstract

Computed tomography (CT) of the head is used worldwide to diagnose neurologic emergencies. However, expertise is required to interpret these scans, and even highly trained experts may miss subtle life-threatening findings. For head CT, a unique challenge is to identify, with perfect or near-perfect sensitivity and very high specificity, often small subtle abnormalities on a multislice cross-sectional (three-dimensional [3D]) imaging modality that is characterized by poor soft tissue contrast, low signal-to-noise using current low radiation-dose protocols, and a high incidence of artifacts. We trained a fully convolutional neural network with 4,396 head CT scans performed at the University of California at San Francisco and affiliated hospitals and compared the algorithm’s performance to that of 4 American Board of Radiology (ABR) certified radiologists on an independent test set of 200 randomly selected head CT scans. Our algorithm demonstrated the highest accuracy to date for this clinical application, with a receiver operating characteristic (ROC) area under the curve (AUC) of 0.991 ± 0.006 for identification of examinations positive for acute intracranial hemorrhage, and also exceeded the performance of 2 of 4 radiologists. We demonstrate an end-to-end network that performs joint classification and segmentation with examination-level classification comparable to experts, in addition to robust localization of abnormalities, including some that are missed by radiologists, both of which are critically important elements for this application.

## Significance

Computed tomography (CT) of the head is the workhorse medical imaging modality used worldwide to diagnose neurologic emergencies. However, these gray scale images are limited by low signal-to-noise, poor contrast, and a high incidence of image artifacts. A unique challenge is to identify tiny subtle abnormalities in a large 3D volume with near-perfect sensitivity. We used a single-stage, end-to-end, fully convolutional neural network to achieve accuracy levels comparable to that of highly trained radiologists, including both identification and localization of abnormalities that are missed by radiologists.

Head computed tomography (CT) is used worldwide to diagnose neurologic emergencies, such as acute traumatic brain injury (TBI), stroke, and aneurysmal hemorrhage. Evaluation for acute intracranial hemorrhage plays a decisive role in the clinical management of these conditions. It is critical for deciding on the need and approach for emergent surgical intervention. It is also essential for allowing the safe administration of thrombolytic therapy in acute ischemic stroke. Since “time is brain,” increased speed and reduced error in these clinical settings would constitute life-saving innovations.

Advances in computer vision techniques, such as deep learning, have demonstrated tremendous potential for extracting clinically important information from medical images. Examples include grading of diabetic retinopathy on retinal fundus photographs (1), detection of metastases in histologic sections of lymph nodes (2), and classification of images of skin cancer (3), with accuracies comparable to or, in some cases, exceeding that of experts. In contrast to these applications, many radiological imaging studies, such as CT and magnetic resonance imaging (MRI), are “cross-sectional,” or three-dimensional (3D), in nature and thus comprised of volumetric stacks of images rather than single images. The 3D nature of such examinations presents an extra challenge. An additional unusual challenge regarding head CT is the need to identify, with perfect or near-perfect sensitivity, often tiny subtle abnormalities occupying ∼100 pixels on noisy, low-contrast images in a large 3D volume that comprises >10^6^ pixels. Finally, although perfect sensitivity at examination-level classification is the most crucial goal, concurrent localization of abnormalities on head CT is also important since physicians will always need to personally visualize and confirm the locations of abnormalities on a head CT examination, in order to judge the need and approach for surgical intervention.

Using a strong pixel-level supervision approach and a relatively small training dataset, we demonstrate an end-to-end network that performs joint classification and segmentation. It demonstrates the highest classification accuracy to date, compared to other deep learning approaches (4–8), and also concurrently localizes these abnormalities. We demonstrate that it identifies many abnormalities missed by experts. In addition, we demonstrate promising results for multiclass hemorrhage segmentation, while preserving accurate detection at the examination level.

## Results

### Main Results.

**Fig. 1.**
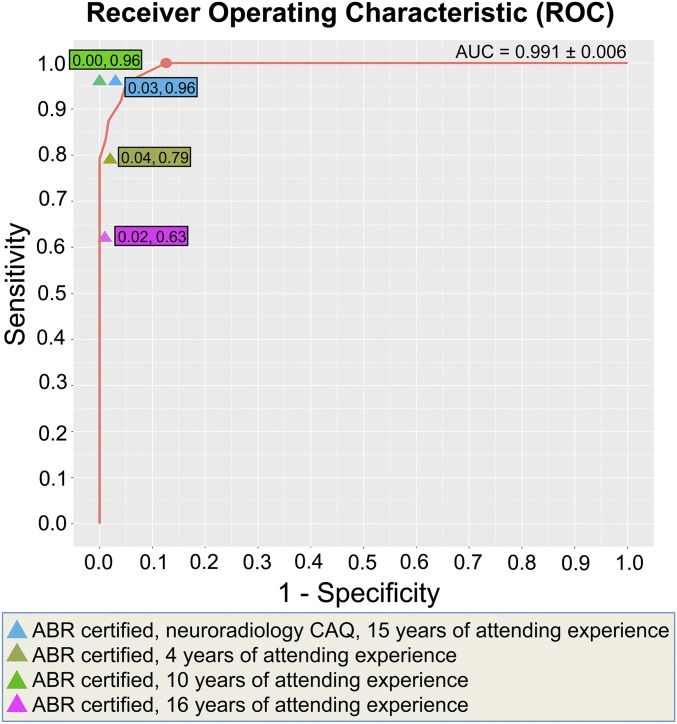
Receiver operating characteristic (ROC) for the deep learning model to predict the presence of acute intracranial hemorrhage on 200 head CT examinations. The algorithm achieved an area under the curve (AUC) of 0.991 ± 0.006 referenced to the gold standard (consensus interpretation of 2 ABR-certified neuroradiologists with a CAQ in neuroradiology). Algorithm performance exceeded that of 2 of 4 American Board of Radiology (ABR)-certified radiologists with attending-level experience ranging from 4 to 16 y. In addition, PatchFCN achieved 100% sensitivity at specificity levels approaching 90%, making this a suitable screening tool for radiologists based on an acceptably low proportion of false positives. The 2 numbers in each color box are the *x* coordinate (1-specificity) and *y* coordinate (sensitivity) for that radiologist’s performance. The salmon-colored circle shows (sensitivity 1.00, specificity 0.87) the highest specificity operating point with perfect sensitivity.

**Fig. 2.**
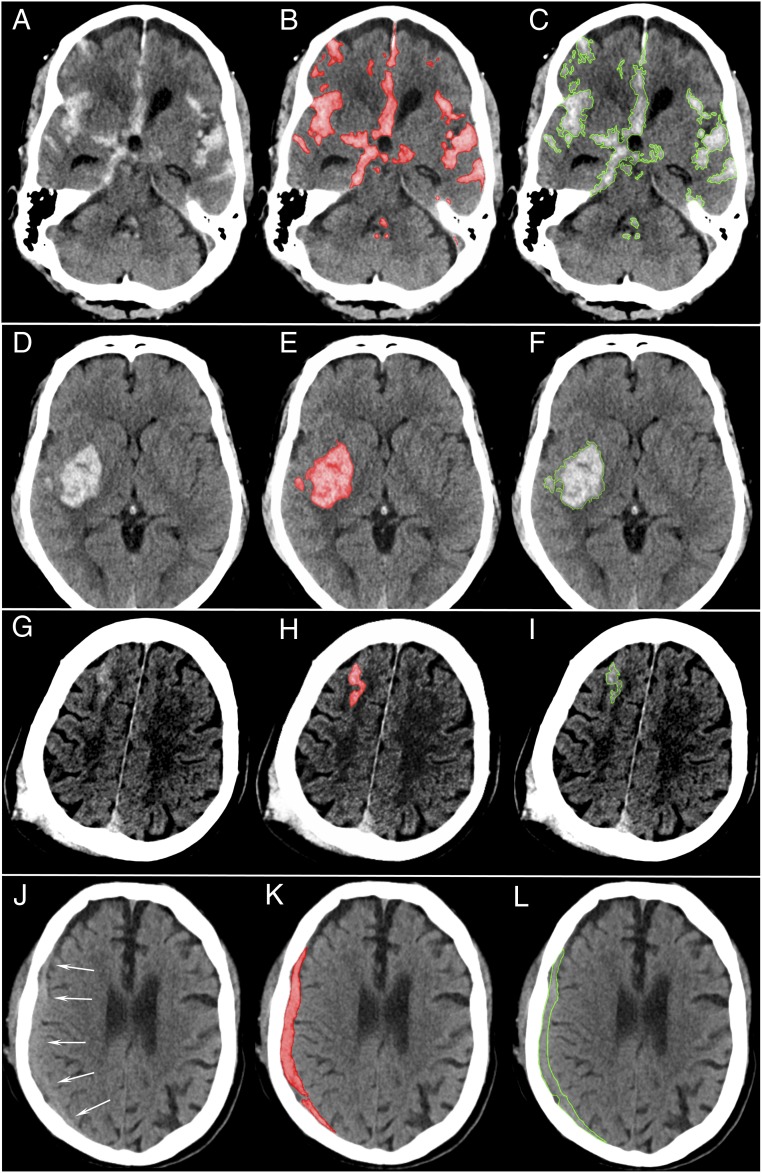
Patch-based fully convolutional neural network (PatchFCN) segmentation of acute intracranial hemorrhage. (*A*–*C*) Subarachnoid hemorrhage (SAH) due to aneurysm rupture. (*D*–*F*) Acute intracerebral hemorrhage. (*G*–*I*) Traumatic SAH (missed by 1 of 4 radiologists) and (*J*–*L*), isodense subdural hematoma (SDH). (*J*–*L*) Acute SDH in the setting of coagulopathy versus subacuted SDH at 2 to several days after injury. The arrows in *J* indicate the border between the SDH and adjacent brain. Because isodense subdural hematomas are not brighter than the adjacent brain parenchyma, radiologists identify these by recognizing the absence of sulci and gyri within the isodense collection. In *J*–*L*, the SDH is detected despite its isodensity to gray matter, showing that the deep learning algorithm does not rely solely on hyperdensity but also uses other features to identify hemorrhage. (*A*, *D*, *G*, and *J*) Original images. (*B*, *E*, *H*, and *K*) Original images with red shading of pixel-level probabilities >0.5 (on a scale of 0 to 1) for hemorrhage, as determined by the PatchFCN; pixels with probability <0.5 were unaltered from the original images. (*C*, *F*, *I*, and *L*) Neuroradiologist’s segmentation of hemorrhage using green outline.

**Fig. 3.**
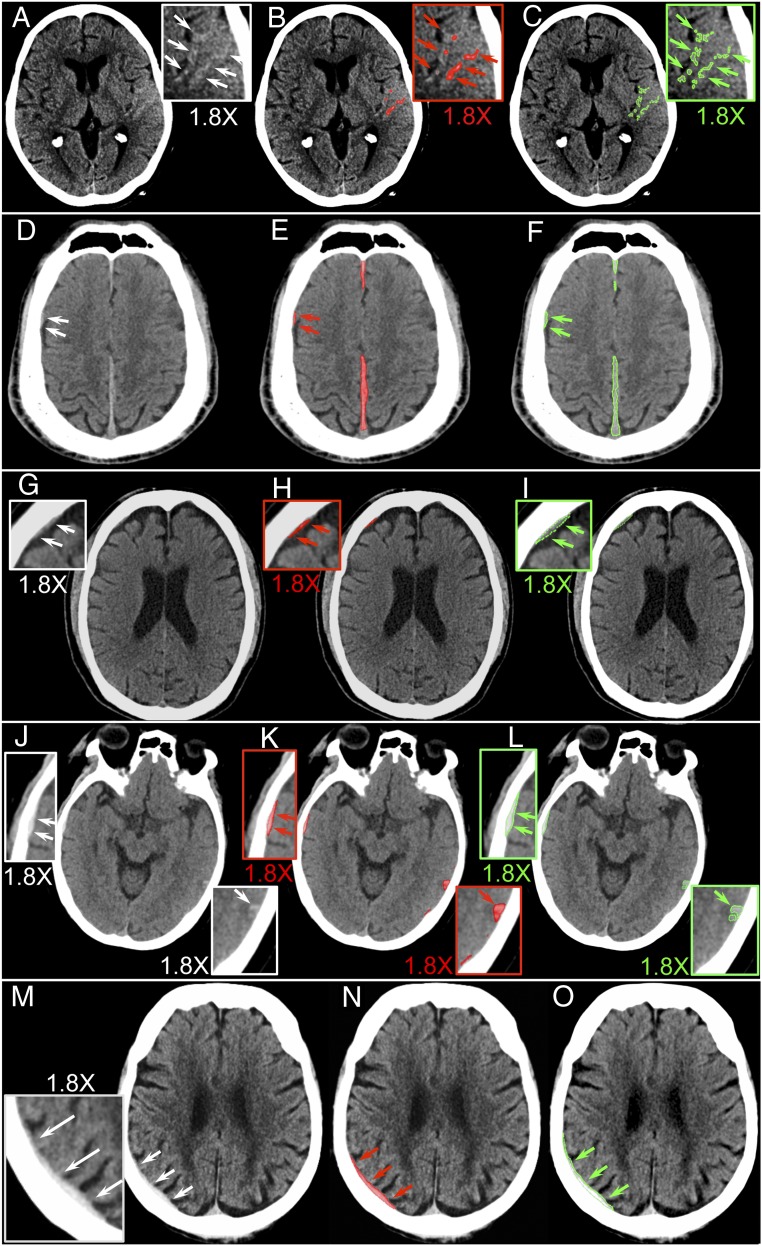
Five cases judged negative by at least 2 of 4 radiologists, but positive for acute hemorrhage by both the algorithm and the gold standard. (*A*–*C*) Small left temporal subarachnoid hemorrhage (SAH), (*D*–*F*) small right posterior frontal and parafalcine subdural hematomas (SDH), (*G*–*I*) small right frontal SDH, and (*J*–*L*) small right temporal epidural hematoma and left posterior temporal contusion were each called negative by 2 of 4 radiologists. (*M*–*O*) Called negative by all 4 radiologists but contained a right parietal SDH identified by both the algorithm and by the gold standard. (*A*, *D*, *G*, *J*, and *M*) Original images. (*B*, *E*, *H*, *K*, and *N*) Algorithmic delineation of hemorrhage with pixel-level probabilities >0.5 colored in red. (*C*, *F*, *I*, *L*, and *O*) Neuroradiologist segmentation of hemorrhage using a green outline. The boxed areas are magnified views of small areas of hemorrhage. Arrows indicate the borders of small or subtle hemorrhages.

Fig. 1 shows that our system patch-based fully convolutional neural network (PatchFCN) performance exceeded that of 2 of 4 American Board of Radiology (ABR)-certified radiologists, with a receiver operating characteristic (ROC) with area under the curve (AUC) of 0.991 ± 0.006 for identification of acute intracranial hemorrhage, referenced to the gold-standard consensus interpretation of 2 ABR-certified neuroradiologists with a Certificate of Added Qualification (CAQ) in neuroradiology. In addition, PatchFCN achieved 100% sensitivity at specificity levels approaching 90%, making this a suitable screening tool with an acceptably low proportion of false positives. Fig. 2 *A*–*L* shows examples of PatchFCN localization of acute intracranial hemorrhage in acute aneurysm rupture, hemorrhagic stroke, subacute traumatic brain injury, and acute traumatic brain injury. Of note, Fig. 2 *J*–*L* shows an isodense subdural hemorrhage and demonstrates that the PatchFCN algorithm cannot rely solely on hyperdensity relative to brain in order to identify acute hemorrhage, but must also use other more subtle features, as do experienced radiologists. Fig. 3 *A*–*O* demonstrates all positive cases in the 200-examination test set that were missed by at least 2 of 4 radiologists.

**Fig. 4.**
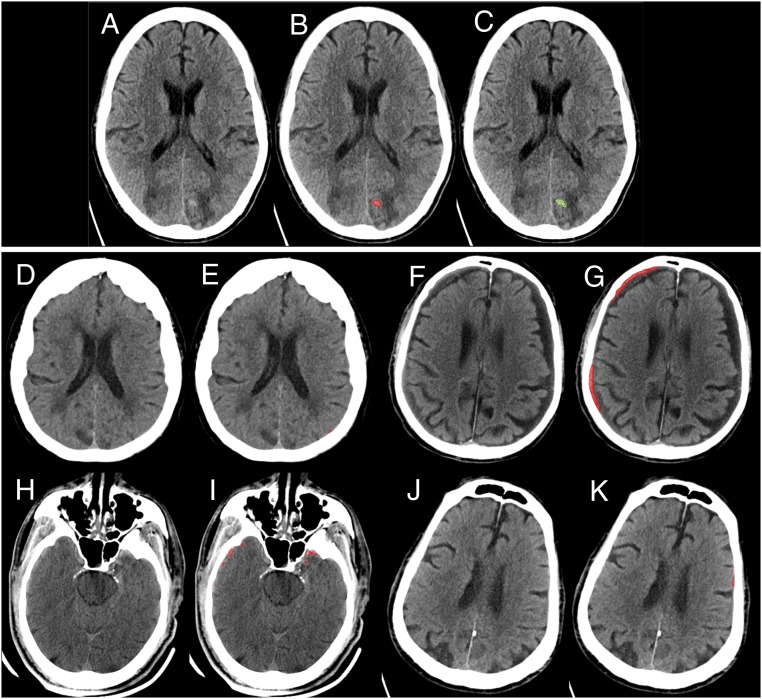
Examples of algorithm “near misses.” As one moves along the algorithm’s ROC curve to sensitivities <1.00, the next discrete operating point occurs at (sensitivity 0.96, specificity 0.98). (*A*–*C*) Initial case “missed” by the algorithm as one moves along the algorithm’s ROC curve from sensitivity 1.00 to the next discrete operating point at sensitivity 0.96. Small areas of faint hyperdensity are present on a background of abnormally hypodense white matter. Original images (*A*), computer algorithm delineation of pixel-level probabilities >0.4 shown in red (*B*), and neuroradiologist segmentation of hemorrhage using a green outline (*C*). We hypothesize that the algorithm’s certainty for this case was borderline because this case resembles negative cases in the training data that demonstrated faint mineralization, resembling hemorrhage, within areas of remote brain infarction. (*D*–*K*) Four false-positive cases at (sensitivity 0.96, specificity 0.98). In *F* and *G*, the algorithm demarcates an area of true intracranial hemorrhage. However, it was designated as a chronic, rather than acute, subdural hematoma in the gold-standard consensus review. *D*, *E*, *J*, and *K* show tiny areas of false-positive areas of hemorrhage delineated by the algorithm on only 1 or 2 images of these examinations. *H* and *I* show peripheral areas of false-positive hemorrhage due to streak artifact and nonlinear partial volume artifact (9) that are common at the level of the skull base.

It is interesting to consider what types of errors are made as one moves along the algorithm’s ROC curve (Fig. 1) toward sensitivities <1.00. At the next discrete operating point, sensitivity 0.96 and specificity 0.98, the single case “missed” by the algorithm is shown in Fig. 4 *A*–*C*. In this case, small areas of faint hyperdensity are present on a background of abnormally hypodense white matter. We hypothesize that the algorithm’s certainty for this case was borderline because this case resembles several negative cases in the training data that demonstrated faint calcifications within areas of remote infarct. The difference is that such cases of remote infarct demonstrate brain volume loss in the areas of calcification while the case in Fig. 4 *A*–*C* does not. This is a subtle distinction that requires a trained human eye or, presumably, in the case of computer algorithms, a large number of cases to “teach” the algorithm to make this distinction. Fig. 4 *D*–*K* shows the 4 false-positive cases at sensitivity 0.96 and specificity 0.98. One of the 4 cases (Fig. 4 *F* and *G*) contains subdural hemorrhage but was designated as “negative” in the gold-standard consensus review since the finding appeared to be chronic rather than acute. The other 3 false-positive cases (Fig. 4 *D*, *E*, and *H*–*K*) demonstrate mostly tiny peripheral false-positive pixels due to a cupping artifact or to a nonlinear partial volume artifact that is common at the level of the skull base (9).

To confirm reproducibility of results, we conducted 4-fold cross-validation experiments. We randomly split the University of California at San Francisco (UCSF)-4.4K training data into 4 subsets. For each of 4 experiments, 3 of 4 of the subsets were used for training and 1 of 4 was held out as a test set. The 4 resulting ROC curves demonstrated AUC values of 0.978 ± 0.003, which were unsurprisingly lower than the AUC of 0.991 based on training on the full UCSF-4.4K set. However, the small SD of 0.003 demonstrates reproducibility of results. Regarding localization accuracy, the algorithm achieved an average Dice coefficient of 0.75 on the 4-fold cross-validation experiments.

### Multiclass Exploratory Study.

**Fig. 5.**
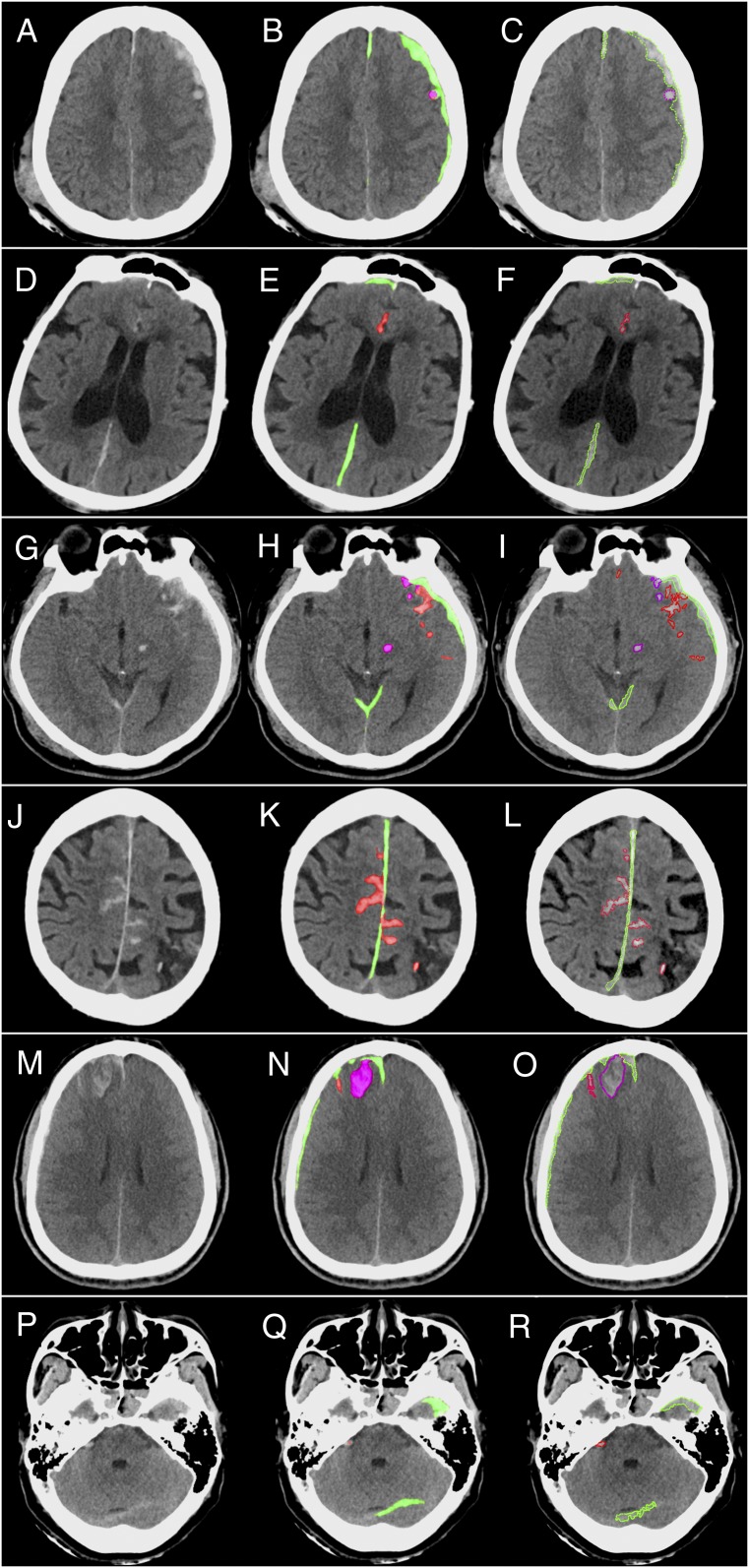
Examples of multiclass segmentation by the algorithm and by an expert. (*A*–*C*) Small left holohemispheric subdural hematoma (SDH, green) and adjacent contusion (purple). (*D*–*F*) Small right frontal and posterior parafalcine SDH and anterior interhemispheric fissure SAH (red). (*G*–*I*) Small bilateral tentorial and left frontotemporal SDH (green) and subjacent contusions (purple) and SAH (red), in addition to shear injury in the left cerebral peduncle (purple). (*J*–*L*) Small parafalcine SDH (green) with surrounding SAH (red). (*M*–*O*) Several small right frontal areas of SDH (green) with subjacent contusion (purple) and SAH (red). (*P*–*R*) Small left tentorial and left anterior temporal SDH (green) and right cerebellopontine angle SAH (red). (*A*, *D*, *G*, *J*, *M*, and *P*) Original images. (*B*, *E*, *H*, *K*, *N*, and *Q*) Algorithmic delineation of hemorrhage with pixel-level probabilities >0.5 colored in red (SAH), green (SDH), and contusion/shear injury (purple). (*C*, *F*, *I*, *L*, *O*, and *R*) Neuroradiologist segmentation of hemorrhage.

Fig. 5 shows examples of multiclass segmentation by the algorithm and by a neuroradiologist. Table 1 shows that PatchFCN achieves competitive examination-level multiclass detection, while maintaining the strong 2-class results on 4-fold cross-validation. The results are reported as mean ± 1 SD. We note that the examination-level prediction of each class (including the combined class) is made independently at the output layer so their results do not depend on each other.

**Table 1.** Examination-level multiclass hemorrhage detection

Class	1	2	3	4	Combined
Hemorrhage types	SDH	EDH	Contusion, ICH, TAI	SAH, IVH	All types
AUC of ROC	0.954 ± 0.010	0.940 ± 0.016	0.934 ± 0.007	0.956 ± 0.006	0.982 ± 0.004

EDH, epidural hematoma; ICH, intracerebral hematoma; IVH, intraventricular hemorrhage; SAH, subarachnoid hemorrhage; SDH, subdural hematoma; TAI, traumatic axonal injury.

## Discussion

We report a deep learning algorithm with accuracy comparable to that of radiologists for the evaluation of acute intracranial hemorrhage on head CT. We show that deep learning can accurately identify diverse and very subtle cases of a major class of pathology on this “workhorse” medical imaging modality. Head CT interpretation is regarded as a core skill in radiology training problems, and the performance bar for this application is accordingly high, with the most skilled readers demonstrating sensitivity/specificity between 0.95 and 1.00.

In this study, we demonstrate, to our knowledge, the highest accuracy levels to date for this application by using a PatchFCN with strong supervision and a relatively small training dataset, compared to prior work relying on weaker supervision using examination- or image-level labels (4–7) or Mask R-CNN (8). We show that FCN with pixel-level supervision is well-suited to this application, in which poorly marginated abnormalities of widely varying sizes and morphologies, such as hemorrhage, need to be both detected and localized. Our approach is fundamentally different from Mask R-CNN (10), which first detects an object and delineates its location using a bounding box and then carries out a pixelwise segmentation within the box. Since hemorrhage is fluid (“stuff,” e.g., water, sky, grass) (11) and takes on highly variable morphologies often without well-defined boundaries separating discrete objects (“things,” e.g., cup, car), semantic segmentation is a simple elegant approach, without the requirements of object detection and region processing associated with Mask R-CNN.

In addition, motivated by the clinical need to identify and localize, in most cases, a very sparse foreground (e.g., examples of hemorrhage in Fig. 3) with high sensitivity, we found the best performance with a PatchFCN that was informed by just the “right” amount of local information (12). Specifically, limitation of the network evaluation of each two-dimensional (2D) image on any single pass to a subset or “patch” of the 2D image for modeling *x*–*y* axes context consistently outperformed evaluation of the entire 2D image on pixel and examination level (12). A reason for this may be that deeper models with a massive number of free parameters may overfit to less relevant distant information in large input images in the setting of a limited dataset size. Similarly, we found, for the current application and dataset size, that a network informed by 3 consecutive images (image under evaluation and “flanking” images immediately superior and inferior) was as accurate for pixel and examination-level classification as a network that employed 5 or more consecutive images, sparing the need for learning even more context with 3D-FCN and avoiding the problem of overfitting to too large a context (12). 3D-FCN takes in the entire 3D volume and was demonstrated to achieve accuracy levels exceeding that of human experts for classification of OCT examinations (13). For the current application, in which a single small localized area of <100 pixels on a single image may represent the sole abnormality in a 3D volumetric stack comprising ∼30 images and >10^6^ pixels, we found that the theoretical advantage of taking in more global context was outweighed by the advantages of 1) forcing the network to consider an intermediate amount of spatial context, both in-plane and in the craniocaudal direction, and 2) larger batch diversity to stabilize training through the use of batch normalization in deep networks (12).

**Fig. 6.**
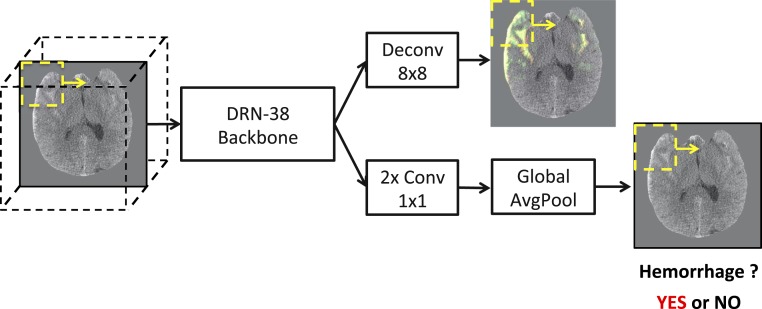
System diagram. Given a head CT stack, we evaluated each frame using a sliding window at inference time. The results were aggregated by averaging at the pixel level (*Top Right* image) where the green shows the prediction and red shows the ground truth annotation. Each frame stacked with its top and bottom neighbors was evaluated by the DRN-38 backbone. Along the top pathway, we applied deconvolution to the top-level features to decode the pixelwise prediction. Along the bottom pathway, we applied 2 convolutions, followed by global average pooling to obtain patchwise classification (*Bottom Right* image). The stack-level score is given by the maximum patch-level score within the stack.

To address the need for both accurate examination-level classification and concurrent localization of abnormalities, we used a single-stage network for joint segmentation and examination-level classification, which enjoys the advantages of 1) only 1 network for both segmentation and examination classification instead of 2 at both training and test time, and 2) significant feature sharing between segmentation and classification networks. In general, it is beneficial to share the representation between correlated tasks, which saves computation and also serves as an effective regularization method (14). Fig. 6 summarizes our hemorrhage detection system architecture.

Further work needs to address the need to maintain high accuracy in the face of data domain shifts across a larger cross-section of CT scanner protocols and hardware at different institutions. Although our dataset includes the 2 largest CT vendors, different institutions use different technical parameters. Algorithm performance needs to be robust to variations in these parameters. Regarding further improvement in algorithm accuracy, additional training data labeled by multiple expert readers and employing more use of ensembles to mitigate random effects, such as random initialization of model weights and random sampling of minibatches, may also slightly enhance accuracy.

The 5 cases judged negative by at least 2 of 4 radiologists, but positive by both the algorithm at sensitivity of 1.0 and the gold standard, admittedly contained very tiny intracranial hemorrhages that are more likely to be stable than to result in significant morbidity or mortality. However, expansion of hemorrhage on any individual case is variable and unpredictable, and it is important to operate at high sensitivities since many patients are taking aspirin or other antiplatelet agents or anticoagulants or may be administered fibrinolytics in the setting of acute stroke. Missed hemorrhages in such cases could have devastating adverse outcomes. Also, aneurysmal subarachnoid bleeding may initially present as tiny “sentinel” hemorrhages that can later have life-threatening consequences when an aneurysm ruptures.

In summary, we demonstrate a deep learning algorithm for detection and localization of acute intracranial hemorrhage on head CT, based on a strong supervision approach and a relatively small training dataset. We show performance that is comparable to highly trained experts. Beyond the key clinical tasks of classification of head CT examinations as positive or negative for abnormalities, PatchFCN will be useful for deriving quantitative biomarkers from CT and other radiological examinations. Rudimentary size measurements for intracranial hemorrhage already play a role in practice guidelines for the management of acute hemorrhagic stroke (ABC/2 method for quantifying intracerebral hematoma) (15, 16), acute aneurysmal subarachnoid hemorrhage (Fisher grade) (17), and acute TBI (Marshall and Rotterdam scores, and criteria for performing decompressive hemicraniectomy) (18-20). However, even these coarse measurements are subjective and can be time-consuming to obtain (21). Improved quantitative information has not been explored due to the impracticality of obtaining these measurements, particularly for poorly marginated, ill-defined abnormalities, such as subarachnoid and multifocal intracranial hemorrhage, that are present on many CT exams. The ability to identify, localize, and quantify features is likely to provide more granular data for research into therapies, prognosis, risk stratification, best treatment practices, and the cost-effectiveness of imaging tests.

## Materials and Methods

### Deep Learning Algorithm

#### Network architecture.

We designed a fully convolutional neural network (FCN) called PatchFCN (12). It is an FCN with modifications selected after exploration of several architectures during the algorithm development phase: 1) To account for context in the craniocaudal direction, it uses 3 input channels consisting of the “flanking” images immediately superior and inferior to the image under evaluation, in order to simulate radiologists’ practice of adjudicating tiny hemorrhages by using contextual information in slices adjacent to the image of interest; 2) to model *x*–*y* axes context, the network evaluation on any single pass is limited to a subset or “patch” of the image, which forces the network to make decisions based on more informative local image information; and 3) to detach the patch prediction from the noisier pixel predictions and to increase patch prediction accuracy, it includes a patch classification branch. The entire system is shown in Fig. 6.

#### Data preprocessing.

The skull and face were removed from CT images using a series of image processing techniques, including thresholding, to identify skull and facial bones, followed by a series of close, open, and fill operations to retain only the intracranial structures. This enhanced privacy of the data as individuals could, in theory, be identified through surface rendering of facial soft tissue pixels present in the original data. It also makes the problem easier for the network as it only needs to model the intracranial structures.

#### Implementation details.

The network backbone architecture was Dilated ResNet 38 (22), and all hyperparameters were developed on the UCSF-4.4K training set described below. We optimized cross-entropy loss with stochastic gradient descent (SGD) and a momentum of 0.99. The learning rate was decreased by 0.1 every 160 epochs. To control class imbalance between positive and negative cases in the training dataset, we sampled 30% of the patches from positive images in each training minibatch and up-weighted the positive pixel loss by a factor of 3. As the multiclass experiments were exploratory in nature, they were performed without any balancing across positive class types. At training time, the backbone and the pixel prediction branch (1 up-convolution layer) were trained at an initial learning rate of 10^−3^ for 400 epochs. Both of these were then fixed, and the patch classification branch (conv + batchnorm + ReLu + conv layers) was trained for 40 epochs. Finally the entire model was jointly fine-tuned for 30 epochs at a learning rate of 5 × 10^−5^. At inference time, adjacent patches were sampled at two-thirds overlap with each other. The pixel predictions in each patch were mapped to image space and averaged to yield the final prediction. The stack classification score was taken as the maximum patch classification score in the stack. The model evaluates each stack within 1 s on average.

#### Multiclass architecture.

We conducted an exploratory study on the multiclass prediction of hemorrhage types at the pixel and examination levels. The model output layers are redesigned for the tasks as follows: 1) The pixel classifier has N + 1, instead of 2, output channels, where N is the number of hemorrhage classes. 2) The stack classification branch has 2(N + 1) outputs for the N hemorrhage classes and the combined positive class. This design is motivated by the observation that the classes are mutually exclusive at the pixel level (i.e., each pixel is a member of only 1 class, or subtype, of hemorrhage) but not at the examination level (i.e., each examination can contain multiple classes of hemorrhage).

### Datasets

#### Training dataset.

All patient data used in this study were collected retrospectively and deidentified, with no need for additional patient contact. Based on US regulation 45 CFR 46.116(d) and the US Food & Drug Administration (FDA) at https://www.fda.gov/media/106587/download, this study satisfied recommended conditions for ethically acceptable waiver of consent due to 1) minimal risk to patients, 2) no adverse effect on the welfare of patients, and 3) the impracticality of contacting very large numbers of subjects for a retrospective study. The study protocol was approved by the UCSF Committee on Human Research.

To develop the algorithm, we used a training set composed of 4,396 head CT scans performed at UCSF and affiliated hospitals (Table 1). This dataset (UCSF-4.4K) consists of 1,131 examinations positive for intracranial hemorrhage and 3,265 negative examinations. The training dataset had a wide spectrum of sizes and types of hemorrhage, as well as of imaging artifacts, and was collected from 4 different CT scanners from 2 major CT vendors (GE Healthcare and Siemens Healthineers) from 2010 to 2017. Each examination consisted of a 3D stack of 27 to 38 transverse 2D images through the head acquired on 64-detector row CT scanners. Pixelwise labels for acute intracranial hemorrhage were verified by 2 ABR-certified radiologists with a CAQ in neuroradiology.

#### Test dataset.

To validate the algorithm, we collected a separate test set of 200 head CT scans performed at the same hospitals in November to December 2017. Table 2 shows the distribution of positive and negative cases across machine types, for both the training and test sets. Although CT scans from GE scanners were characterized by a higher proportion of positive cases than were CT scans from Siemens scanners in the training dataset, the reverse was true for cases in the test set. If the algorithm “cheated” to predict the presence or absence of hemorrhage by using image features to recognize the CT manufacturer that produced the image, this would have had a tendency to degrade rather than enhance the algorithm’s performance on the test set. This obviated the possibility that the strong performance of the algorithm could be attributable to subtle differences in the images based on CT manufacturer alone, rather than the presence or absence of hemorrhage.

**Table 2.** Training and test datasets

Dataset	Result	GE	Siemens	Neurologica	Total
Training	Positive	821	310	0	1,131
	Negative	768	2,497	0	3,265
Test	Positive	0	25	0	25
	Negative	14	160	1	175

In formulating the test set, we aimed for an overall 10% to 15% positive rate for acute intracranial hemorrhage that approaches the positive head CT rate in many busy acute-care hospitals. We also wished to evaluate the algorithm on the initial head CT examination only and to exclude follow-up head CT examinations performed during the same hospitalization following neurosurgical interventions, such as hemicraniectomy or craniotomy. We also aimed to include within the test set a substantial number of positive examinations that would include a diverse spectrum of possible intracranial hemorrhage patterns, while maintaining an overall low positive head CT rate that would simulate observed rates in current clinical practice. We needed to control the overall test set size, such that each adjudicating radiologist could interpret the entire set of 200 head CT examinations within a total of 5 d when working at an average clinical pace. Finally, we wished to minimize selection bias in the process of selecting cases for the test set. To accomplish these goals for the test set, we used the following approach. The examinations were identified from the Radiology Information System (RIS) Structured Query Language (SQL) database. Using the RIS, we randomly selected 150 head CT examinations ordered from November to December 2018 that excluded reference to a prior craniectomy or craniotomy and for which no prior or follow-up head CT examination was found for that patient during the same hospitalization. We also randomly selected 50 head CT examinations with no reference to prior craniectomy or craniotomy and no prior head CT examination during the same hospitalization, but with at least 1 follow-up head CT scan performed during the same hospitalization. Since most CT scans with no follow-up CT scan during the same hospitalization are negative for an acute intracranial abnormality, while many (but not all) CT scans with at least 1 follow-up CT scan performed during the same hospitalization contain a significant acute intracranial finding, we estimated that this strategy would yield an overall 10% to 15% proportion of positive head CT examinations for acute intracranial hemorrhage, while avoiding the need to view the actual images. Using this approach, the actual test set of 200 examinations contained 25 positive and 175 negative examinations for acute intracranial hemorrhage, for an overall 12.5% positive rate that approximates the observed positive head CT rate in many hospitals. The skull stripping algorithm failed on one head CT examination, which was replaced by another examination from the same time period using the same approach. The test set did contain a larger proportion of Siemens CT examinations compared to the CT vendor distribution in the UCSF-4.4K training dataset, owing to the larger number of head CT examinations performed on Siemens CT scanners as part of the acute head CT workflow in place at Zuckerberg San Francisco General Hospital and Trauma Center (ZSFG) during the November to December 2017 time period.

#### Multiclass data.

To explore the potential of PatchFCN in multiclass setting, we collected an expanded set of multiclass hemorrhage data that comprises 4,766 scans from GE and Siemens scanners. Each pixel was labeled according to the hemorrhage types shown in Table 3. The pixel and examination ratios of each hemorrhage type indicate the proportion of positive pixels/examinations for each type of hemorrhage within the multiclass dataset. Note that positive-class pixels are extremely rare compared to negative pixels. The scarcity of foreground pixels in conjunction with low-contrast noisy images makes both pixel and examination-level prediction challenging.

**Table 3.** Multiclass exploratory data

Class	0	1	2	3	4
Hemorrhage types	None	SDH	EDH	Contusion, ICH, TAI	SAH, IVH
Pixel ratio	0.996	3.5 × 10^−3^	3.2 × 10^−4^	2.2 × 10^−5^	7.1 × 10^−4^
Examination ratio	0.686	0.196	0.026	0.152	0.232

EDH, epidural hematoma; ICH, intracerebral hematoma; IVH, intraventricular hemorrhage; SAH, subarachnoid hemorrhage; SDH, subdural hematoma; TAI, traumatic axonal injury.

#### Data availability.

The data used to train and test the machine learning models are administered by the University of California (California Code Regs. title. 22 Section 70751). The dataset in its entirety is not currently publicly available, but a subset may be available for research, subject to approval of the University of California.

#### Code availability.

The deep learning algorithms were developed in PyTorch based on a publicly available codebase on Github: https://github.com/fyu/drn. Although the full code used for experiments described here is not currently publicly available, the description of the network architecture in *Materials and Methods* contains all details needed to reproduce the results. We can provide our source code upon request, subject to approval by the University of California.

### Evaluation of Deep Learning Algorithm Performance and Comparison to Radiologists

#### Evaluation of model performance.

To evaluate model performance, the deep learning algorithm was executed exactly once on the test set of 200 CT examinations, with no adjustment of hyperparameters that had been selected during the algorithm development phase. This excluded the possibility of any overfitting to the test data so that the reported performance should match the model’s true performance very well. For each scan in the test dataset consisting of 200 CT examinations, the algorithm indicates both pixel-level and examination-level probabilities (continuous from 0 to 1) for the presence of intracranial hemorrhage. Although some patients underwent 2 or more head CT examinations during the same hospitalization, it was ensured that each patient appeared at most once in either the training set or the test set, but not in both.

We calculated the receiver operating characteristic (ROC) for the deep learning algorithm to identify the presence of acute intracranial hemorrhage on each CT examination, compared to the “gold standard.” The gold standard for interpretation of all 200 CT scans in the test set as positive or negative for acute intracranial hemorrhage consisted of a careful consensus interpretation by 2 ABR-certified neuroradiologists with a CAQ in neuroradiology, one with 15 y and the other with 10 y of attending-level experience in interpretation of head CT examinations.

#### Comparison to radiologists.

Four ABR-certified practicing radiologists each reviewed the 200 CT examinations in the test set. One radiologist had 2 y of subspecialty fellowship training and a CAQ in neuroradiology, with 15 y of attending neuroradiologist experience. The others had 4, 10, and 16 y of experience in private and/or academic general radiology practice, including interpretation of head CT. Radiologists were asked to indicate whether each scan was more likely positive or more likely negative for acute intracranial hemorrhage, a binary decision, in contrast to the continuous probability for hemorrhage provided for each examination by the algorithm. Radiologists’ time to evaluate each scan was not limited. Radiologists were instructed to interpret all CT scans carefully, using conventions, such as the duration of time spent on each scan, and level of care in interpreting each scan, that would be consistent with US standard-of-care clinical practice. Radiologists were able to return to prior CT scans and to modify their interpretations of examinations they had seen earlier in the dataset. Radiologists were not aware of the overall ratio of positive to negative CT examinations. We calculated the sensitivity and specificity of each radiologist to detect whether or not there was acute intracranial hemorrhage on each CT examination, compared to the gold standard.
